# Associations Between Types of Physical Activity and Mental Health Among Korean Adolescents: A Cross-Sectional Study Using Data from the 21st Korea Youth Risk Behavior Survey (2025)

**DOI:** 10.3390/bs16101867

**Published:** 2026-10-10

**Authors:** Sung-Ho Hwang, Su-Yeon Roh, Geun-Kook Kim

**Affiliations:** 1 Department of Sports Medicine, Shinhan University, Uijeongbu 11644, Republic of Korea; 1067hsh@shinhan.ac.kr; 2 Department of Exercise Rehabilitation, Gachon University, Seongnam 13120, Republic of Korea; 3 Department of Sports Rehabilitation, Jaeneung University, Incheon 22573, Republic of Korea

**Keywords:** adolescents, physical activity, mental health, suicidal behavior, Korea youth risk behavior survey

## Abstract

Adolescence is critical for developing physical activity habits and maintaining mental health. Using data from the 21st Korea Youth Risk Behavior Survey (2025), this cross-sectional study examined the associations between physical activity and mental health in Korean adolescents. The sample included 52,618 middle and high school students. Physical activity included ≥60 min/day of physical activity, vigorous physical activity, muscle-strengthening exercise, active transportation, and physical education class participation. Mental health indicators included stress perception, depressive symptoms, suicidal ideation, suicide plans, and suicide attempts; stress perception was examined descriptively, whereas depressive symptoms and suicide-related indicators were evaluated as outcomes in the multivariable analyses. Analyses accounted for the complex survey design, including sampling weights, stratification, and clustering. Survey-adjusted tests and complex-sample multivariable logistic regression were performed, adjusting for sex, grade, academic achievement, household economic status, living arrangement, and weight status. Vigorous physical activity and physical education participation were significantly associated with all four mental health outcomes. Compared with no vigorous physical activity, participation on 1–2 days was associated with lower odds of suicidal ideation (aOR = 0.875, 95% CI: 0.811–0.944), suicide plans (aOR = 0.810, 95% CI: 0.720–0.912), and suicide attempts (aOR = 0.803, 95% CI: 0.688–0.937). Physical education twice per week was associated with lower odds of all four outcomes (aORs = 0.737–0.927). In contrast, several frequency categories of ≥60 min/day of physical activity and muscle-strengthening exercise were associated with higher odds of depressive symptoms. These findings suggest that associations between physical activity and mental health vary by activity type and mental health outcomes.

## 1. Introduction

Adolescence is a critical developmental period characterized by rapid emotional and social changes and physical growth. Mental health during this period may have long-term implications for academic performance, interpersonal relationships, and health outcomes in adulthood (Agnafors et al., 2021). In particular, stress and depressive symptoms may be associated with suicidal ideation, suicide plans, and suicide attempts, underscoring the importance of early identification of mental health problems and their associated factors among adolescents. According to the 21st Korea Youth Risk Behavior Survey (KDCA, 2025), the prevalence of stress perception was 32.9% among male students and 50.3% among female students, whereas the prevalence of depressive symptoms was 21.7% and 29.9%, respectively. These findings indicate that a substantial proportion of Korean adolescents experience stress and depressive symptoms, with female students exhibiting a greater burden of mental health problems (KDCA, 2025).

Adolescent mental health may be associated with not only individual psychological characteristics but also various sociodemographic factors, including sex, grade level, living arrangement, academic achievement, and household economic status. As students advance to higher grade levels, they may experience increasing pressure related to academic demands and future career decisions. Adolescents who do not live with their family or experience economic difficulties may also have limited access to emotional support and health-related resources (Gewirtz O’Brien et al., 2020). Academic pressure has been identified as an important factor associated with depression, anxiety, and suicide-related problems among adolescents (Steare et al., 2023). Moreover, studies on Korean adolescents reported significant associations between economic status and changes in lifestyle behaviors and stress perception, depressive symptoms, and suicidal ideation (Kang et al., 2024; J.-A. Park, 2025; Choi & Kim, 2026). Therefore, understanding adolescent mental health requires consideration of differences in health and mental health indicators according to general characteristics, in addition to physical activity.

Physical activity is an important health behavior that may support not only physical growth and fitness but also mental health and cognitive function among adolescents. Physical fitness has also been associated with academic achievement among secondary school students (Canli et al., 2024). The World Health Organization recommends that children and adolescents engage in an average of at least 60 min per day of moderate-to-vigorous-intensity, mostly aerobic, physical activity across the week and incorporate vigorous-intensity aerobic activities and activities that strengthen muscles and bones at least three days per week (World Health Organization, 2020). However, in 2025, only 24.5% of male and 8.5% of female Korean adolescents engaged in at least 60 min of physical activity per day on five or more days per week. This recommendation differs from the frequency-based indicator used in this study, which reflects the number of days with ≥60 min of physical activity. Similarly, the proportions engaging in muscle-strengthening exercises at least three days per week were 37.7% and 10.3%, respectively, indicating a substantial sex difference (KDCA, 2025). These findings suggest that physical activity participation among Korean adolescents remains insufficient and that opportunities for participation may vary according to individual and social characteristics.

Previous studies reported that higher levels of physical activity are associated with a reduced risk of depression among adolescents (Panza et al., 2020), and exercise interventions demonstrated significant effects on reducing depressive symptoms in this population (Wang et al., 2022). Physical activity may be associated with mental health through physiological pathways, such as improvements in physical fitness and sleep quality, and psychosocial pathways, including enhanced self-efficacy, self-esteem, social relationships, and emotional regulation (Rodriguez-Ayllon et al., 2019; Hou et al., 2025). Furthermore, although physical activity has been reported to be associated with lower levels of suicidal ideation (Vancampfort et al., 2018; Kim et al., 2024), a meta-analysis found that exercise interventions did not significantly reduce suicidal ideation or suicidal behavior (Fabiano et al., 2023). Therefore, it cannot be assumed that physical activity is consistently associated with all mental health indicators in the same direction.

These inconsistencies may be partly attributable to the fact that previous studies assessed overall physical activity without distinguishing between its intensity, purpose, or context. Physical activity for ≥60 min/day and vigorous physical activity reflect the amount and intensity of activity, whereas muscle-strengthening exercise represents a specific type of activity, and active transportation reflects the mode of travel incorporated into daily life. Unlike structured exercise, active transportation is embedded in daily routines and may be influenced by environmental and school-related conditions. Physical education classes are conducted with peers in a structured school setting and, therefore, have social and educational characteristics that differ from those of general leisure-time physical activity. Korean studies suggested that participation in physical education classes may be associated with stress perception and suicidal ideation (S. Park et al., 2020) and that associations with anxiety among adolescents may vary according to the type of physical activity (Shin & Lee, 2024). A recent study also examined associations between different types of physical activity and suicidal behaviors among Korean adolescents (C.-Y. Lee & Choi, 2026). Nevertheless, few Korean studies have simultaneously considered physical activity for ≥60 min/day, vigorous physical activity, muscle-strengthening exercise, active transportation, and participation in physical education classes and compared their associations with multiple mental health indicators ranging from depressive symptoms to suicidal ideation, suicide plans, and suicide attempts.

Therefore, this study aimed to examine the associations of general characteristics and physical activity with mental health status among Korean middle and high school students using data from the 21st Korea Youth Risk Behavior Survey (KDCA, 2025). Specifically, this study aimed to (1) examine differences in self-rated health, body perception, stress perception, and sleep recovery according to general characteristics; (2) compare the distributions of depressive symptoms, suicidal ideation, suicide plans, and suicide attempts according to grade level and physical activity frequency; and (3) examine the adjusted associations between each type of physical activity and depressive symptoms, suicidal ideation, suicide plans, and suicide attempts, accounting for sociodemographic characteristics and weight status. Given the mixed findings reported in previous studies, we hypothesized that the associations with mental health outcomes would vary according to the type and frequency of physical activity rather than showing a uniform pattern across all physical activity domains. The findings of this study are expected to provide fundamental evidence for understanding the relationships between specific types of physical activity and adolescent mental health.

## 2. Materials and Methods

### 2.1. Study Design and Participants

This study was a cross-sectional secondary data analysis using raw data from the 21st Korea Youth Risk Behavior Survey (KDCA, 2025), jointly conducted by the Ministry of Education and the Korea Disease Control and Prevention Agency. The Korea Youth Risk Behavior Survey is an annually conducted, government-approved statistical survey (Approval No. 117058) designed to assess health behaviors among Korean adolescents and provide fundamental data for the development of health policies. The target population consisted of students enrolled in grades 7–12 nationwide. Using stratified cluster sampling, 2400 classes from 800 middle and high schools across Korea were selected for the survey.

The 21st survey was conducted from 9 June–11 July 2025, during regular school hours, using an anonymous, self-administered online questionnaire completed on mobile devices. Of the 57,682 students selected for the survey, 54,170 (93.9%) participated in the study. Among the respondents, missing data were identified for height (*n* = 1535), weight (*n* = 1535), academic achievement (*n* = 6), household economic status (*n* = 12), and living arrangement (*n* = 18). Because missingness overlapped across variables, these counts were not mutually exclusive. After accounting for overlapping missing data, 1552 unique participants (2.9%) had missing data for at least one of these variables and were excluded using a complete-case approach. Accordingly, data from 52,618 participants (97.1%) with complete data for the variables required for the present analysis were included in the final analysis.

### 2.2. Measured Variables

#### 2.2.1. General Characteristics

Participants’ general characteristics included sex, grade level, living arrangement, academic achievement, and household economic status. Sex was categorized as male or female, and grade level was classified into grades 7, 8, and 9 for middle school and grades 10, 11, and 12 for high school. For analyses by school level, students in grades 7–9 were categorized as middle school students, whereas those in grades 10–12 were categorized as high school students.

Living arrangements were classified into five categories: living with family, living with relatives, living in a boarding house or independently, living in a dormitory, or living in a residential care facility. Academic achievement and household economic status were based on the participants’ self-reported assessments and were each classified into five categories: high, upper-middle, middle, lower-middle, and low. All the variables were analyzed according to the response categories specified in the 21st Korea Youth Risk Behavior Survey.

#### 2.2.2. Weight Status

Weight status was classified based on body mass index (BMI), which was calculated using self-reported height and weight. BMI was calculated as body weight in kilograms divided by height in meters squared (kg/m^2^). Weight status was classified according to sex- and age-specific BMI percentiles from the 2017 Korean National Growth Charts. Participants were categorized as underweight (<5th percentile), normal weight (≥5th and <85th percentile), overweight (≥85th and <95th percentile), or obese (≥95th percentile).

#### 2.2.3. Physical Activity

Physical activity was assessed using five indicators: ≥60 min of physical activity, vigorous physical activity, muscle-strengthening exercise, active transportation to and from school, and participation in physical education classes. ≥60 min of physical activity was assessed using the question, “During the past 7 days, on how many days did you engage in physical activity for a total of at least 60 min per day, regardless of the type of activity, that increased your heart rate or made you breathe harder than usual?” Vigorous physical activity was assessed using the question, “During the past 7 days, on how many days did you engage in vigorous physical activity that made you breathe hard or sweat?” Muscle-strengthening exercise was assessed using the question, “During the past 7 days, on how many days did you perform exercises to strengthen your muscles, such as push-ups, sit-ups, weightlifting, dumbbell exercises, pull-ups, or parallel-bar exercises?”

Active transportation to and from school was assessed based on the number of days during the past seven days on which the participants walked or rode a bicycle when traveling to or from school or private educational institutes. Participation in physical education classes was assessed based on the number of times the participants actively engaged in exercise on the playground or in the gymnasium during school physical education classes over the past seven days.

≥60 min of physical activity, vigorous physical activity, muscle-strengthening exercise, and active transportation were each recategorized into four groups: 0 days, 1–2 days/week, 3–4 days/week, and 5–7 days/week. Participation in physical education classes was categorized as 0 times, 1 time/week, 2 times/week, and ≥3 times/week.

#### 2.2.4. Self-Rated Health

Self-rated health, body perception, stress perception, and sleep recovery were included as descriptive health-related indicators and were not treated as primary mental health outcomes or confounding variables in the multivariable analyses. Self-rated health was assessed using a single question: “How would you rate your health in general?” Responses were classified into five categories: “very healthy,” “healthy,” “average,” “unhealthy,” and “very unhealthy”. For analysis, responses were dichotomized as positive self-rated health (“very healthy” or “healthy”) and other responses (“average,” “unhealthy,” or “very unhealthy”).

#### 2.2.5. Body Perception

Body perception was assessed using a single question: “How do you perceive your body shape?” Responses were classified into five categories: “very thin,” “slightly thin,” “average,” “slightly overweight,” and “very overweight.” For analysis, responses were dichotomized as overweight body perception (“slightly overweight” or “very overweight”) and other responses (“very thin,” “slightly thin,” or “average”).

#### 2.2.6. Stress Perception

Stress perception was assessed using a single question: “How much stress do you usually experience?” Responses were classified into five categories: “very much,” “a lot,” “some,” “not much,” and “not at all.” For analysis, responses were dichotomized as perceived stress (“very much” or “a lot”) and other responses (“some,” “not much,” or “not at all”).

#### 2.2.7. Sleep Recovery

Sleep recovery was assessed using a single question: “During the past 7 days, do you think the amount of sleep you obtained was sufficient to recover from fatigue?” Responses were classified into five categories: “very sufficient,” “sufficient,” “moderate,” “insufficient,” and “very insufficient.” For analysis, responses were dichotomized as sufficient sleep recovery (“very sufficient” or “sufficient”) and other responses (“moderate,” “insufficient,” or “very insufficient”).

#### 2.2.8. Mental Health

Mental health was assessed using four indicators: depressive symptoms, suicidal ideation, suicide plans, and suicide attempts. Depressive symptoms were assessed using the question, “During the past 12 months, have you ever felt so sad or hopeless for two consecutive weeks that you stopped doing your usual activities?” Suicidal ideation was assessed using the question, “During the past 12 months, have you ever seriously considered suicide?” Suicide plans were assessed using the question, “During the past 12 months, have you ever made a specific plan to commit suicide?” Suicide attempts were assessed using the question, “During the past 12 months, have you ever attempted suicide?” Responses to each item were analyzed as dichotomous variables (“yes” or “no”). These single-item indicators were derived from the standardized Korea Youth Risk Behavior Survey questionnaire and were interpreted as survey-based indicators rather than clinical diagnostic measures.

### 2.3. Statistical Analysis

The collected data were analyzed using IBM SPSS Statistics version 28.0 (IBM Corp., Armonk, NY, USA). Frequency analyses were performed to examine the distributions of participants’ general characteristics, physical activity, and mental health status, and the results were presented as unweighted frequencies and survey-weighted percentages. All analyses accounted for the complex survey design of the Korea Youth Risk Behavior Survey by incorporating sampling weights, stratification, and clustering.

Complex-sample crosstabulation with Rao–Scott adjusted tests was performed to examine differences in self-rated health, body perception, stress perception, and sleep recovery according to general characteristics. The same survey-adjusted approach was used to examine differences in depressive symptoms, suicidal ideation, suicide plans, and suicide attempts according to grade level and physical activity frequency.

To examine the adjusted associations between physical activity and mental health outcomes, separate complex-sample multivariable logistic regression models were performed for each physical activity type and mental health outcome. Each physical activity domain was examined in a separate model and was not mutually adjusted for the other physical activity domains. Each physical activity variable was entered as a categorical exposure, with no participation as the reference category, and the models were adjusted for sex, grade, academic achievement, household economic status, living arrangement, and weight status. Adjusted odds ratios (aORs) and 95% confidence intervals (CIs) were reported. All statistical tests were two-tailed, and statistical significance was set at *p* < 0.05. Given the number of comparisons, these analyses were considered exploratory, and findings were interpreted based on the magnitude, 95% CIs, direction, and consistency of the associations rather than statistical significance alone. Statistically significant overall tests were distinguished from significant individual frequency-category estimates in the interpretation of the results.

## 3. Results

### 3.1. Participants’ General Characteristics

The participants’ general characteristics are presented in Table 1. A total of 52,618 participants were included in the analysis: 27,018 male students (51.3%) and 25,600 female students (48.7%). Regarding grade level, grade 7 students accounted for the largest proportion, with 9433 (17.5%), followed by grade 8 with 9167 (17.0%), grade 9 with 8951 (16.8%), grade 10 with 8523 (15.9%), grade 11 with 8360 (16.1%), and grade 12 with 8184 (16.6%).

Regarding living arrangements, most participants lived with their families (*n* = 50,065, 95.7%), followed by those living in dormitories (*n* = 1830, 2.8%), living in boarding houses or independently (*n* = 293, 0.6%), living with relatives (*n* = 269, 0.5%), and living in residential care facilities (*n* = 161, 0.4%). Regarding weight status, normal weight was the most prevalent category (*n* = 37,074, 71.1%), followed by obesity (*n* = 6897, 12.6%), overweight (*n* = 4974, 9.2%), and underweight (*n* = 3673, 7.1%).

Regarding academic achievement, the middle category accounted for the largest proportion (*n* = 15,286, 28.9%), followed by upper-middle (*n* = 13,605, 25.9%), lower-middle (*n* = 12,433, 23.4%), high (*n* = 6297, 12.2%), and low (*n* = 4997, 9.6%). Regarding household economic status, the middle category was the most prevalent (*n* = 24,583, 46.0%), followed by upper-middle (*n* = 16,587, 32.0%), high (*n* = 5703, 11.3%), lower-middle (*n* = 4834, 8.9%), and low (*n* = 911, 1.8%).

### 3.2. Differences in Health-Related Indicators According to General Characteristics

Differences in health-related indicators according to the participants’ general characteristics are presented in Table 2. Survey-weighted analyses showed significant differences across grade levels in positive self-rated health, overweight body perception, perceived stress, and sufficient sleep recovery (all *p* < 0.001). The prevalence of positive self-rated health and sufficient sleep recovery generally decreased with increasing grade level, whereas perceived stress tended to increase. Overweight body perception showed relatively small differences across grade levels.

According to living arrangement, significant differences were observed only in sufficient sleep recovery (adjusted *F* = 10.437, *p* < 0.001). Differences in positive self-rated health (adjusted *F* = 1.684, *p* = 0.159), overweight body perception (adjusted *F* = 1.267, *p* = 0.282), and perceived stress (adjusted *F* = 2.600, *p* = 0.054) were not statistically significant.

All four health-related indicators differed significantly according to weight status (all *p* < 0.001). Positive self-rated health was most prevalent among participants with normal weight (71.8%) and least prevalent among those with obesity (55.7%). Overweight body perception increased markedly across weight-status categories, from 0.8% among underweight participants to 96.6% among those with obesity. Perceived stress and sufficient sleep recovery also differed significantly according to weight status.

Significant differences were observed across academic achievement levels for all four health-related indicators (all *p* < 0.001). Lower academic achievement was generally associated with a lower prevalence of positive self-rated health and sufficient sleep recovery and a higher prevalence of overweight body perception and perceived stress. Similarly, all four indicators differed significantly according to household economic status (all *p* < 0.001). As household economic status decreased, the prevalence of positive self-rated health and sufficient sleep recovery decreased, whereas overweight body perception and perceived stress generally increased.

### 3.3. Differences in Mental Health According to Grade Level

Differences in mental health outcomes according to grade level are presented in Table 3. Survey-weighted analyses showed significant differences across grade levels in depressive symptoms (adjusted *F* = 7.496, *p* < 0.001), suicidal ideation (adjusted *F* = 16.242, *p* < 0.001), suicide plans (adjusted *F* = 9.136, *p* < 0.001), and suicide attempts (adjusted *F* = 4.581, *p* < 0.001). The prevalence of depressive symptoms ranged from 23.8% to 27.5% across grades and was highest among Grade 9 students. Suicidal ideation and suicide plans were also most prevalent among Grade 9 students (13.6% and 5.1%, respectively), although the prevalence of suicide plans was identical among Grade 8 students (5.1%). Suicide attempts were most prevalent among Grade 8 students (2.9%) and least prevalent among Grade 12 students (1.9%). Overall, suicidal ideation, suicide plans, and suicide attempts tended to be more prevalent in Grades 7–9 than in Grades 10–12.

### 3.4. Associations Between Physical Activity Frequency and Mental Health Status

The associations between physical activity frequency and mental health status are presented in Table 4. The frequency of ≥60 min of physical activity was significantly associated with depressive symptoms (adjusted *F* = 4.329, *p* = 0.005) and suicidal ideation (adjusted *F* = 7.420, *p* < 0.001), whereas no significant associations were observed with suicide plans (adjusted *F* = 1.034, *p* = 0.376) or suicide attempts (adjusted *F* = 2.144, *p* = 0.093). The prevalence of suicidal ideation was highest among participants reporting no ≥60 min physical activity (12.4%) and ranged from 10.6% to 11.1% among those reporting one or more days per week.

The frequency of vigorous physical activity was significantly associated with all four mental health outcomes: depressive symptoms (adjusted *F* = 3.162, *p* = 0.024), suicidal ideation (adjusted *F* = 18.727, *p* < 0.001), suicide plans (adjusted *F* = 8.313, *p* < 0.001), and suicide attempts (adjusted *F* = 3.868, *p* = 0.009). Participants reporting no vigorous physical activity had the highest prevalence of suicidal ideation (13.0%) and suicide plans (4.9%), whereas depressive symptoms and suicide attempts did not show a consistent pattern across frequency categories.

The frequency of muscle-strengthening exercise was significantly associated with suicidal ideation (adjusted *F* = 11.104, *p* < 0.001) and suicide attempts (adjusted *F* = 4.074, *p* = 0.007), but not with depressive symptoms (adjusted *F* = 0.837, *p* = 0.473) or suicide plans (adjusted *F* = 0.878, *p* = 0.451). Suicidal ideation was most prevalent among participants reporting no muscle-strengthening exercise (12.2%) and least prevalent among those reporting 5–7 days per week (9.5%).

The frequency of active transportation was significantly associated with depressive symptoms (adjusted *F* = 9.978, *p* < 0.001), suicide plans (adjusted *F* = 4.162, *p* = 0.006), and suicide attempts (adjusted F = 8.926, *p* < 0.001), whereas the association with suicidal ideation was not statistically significant (adjusted *F* = 2.459, *p* = 0.062). Participants reporting active transportation on 5–7 days per week had the lowest prevalence of depressive symptoms (24.3%), suicide plans (3.9%), and suicide attempts (2.2%).

The frequency of participation in physical education classes was significantly associated with depressive symptoms (adjusted *F* = 6.676, *p* < 0.001), suicidal ideation (adjusted *F* = 12.879, *p* < 0.001), suicide plans (adjusted *F* = 5.283, *p* = 0.001), and suicide attempts (adjusted *F* = 6.614, *p* < 0.001). Participants reporting no participation in physical education classes had the highest prevalence of depressive symptoms (27.3%), suicidal ideation (13.6%), suicide plans (5.0%), and suicide attempts (3.1%).

### 3.5. Adjusted Associations Between Physical Activity Frequency and Mental Health Outcomes

The adjusted associations between physical activity frequency and mental health outcomes are presented in Table 5. After adjustment for sex, grade, academic achievement, household economic status, living arrangement, and weight status, the associations differed according to the type of physical activity and mental health outcome. In the overall tests across frequency categories, the frequency of ≥60 min of physical activity was significantly associated with depressive symptoms (Wald F = 26.489, *p* < 0.001) and suicide attempts (Wald F = 2.975, *p* = 0.031). At the individual frequency-category level, compared with no participation, the odds of depressive symptoms were higher at 1–2 days (aOR = 1.140, 95% CI: 1.080–1.203), 3–4 days (aOR = 1.231, 95% CI: 1.155–1.312), and 5–7 days (aOR = 1.329, 95% CI: 1.241–1.423).

The overall tests for vigorous physical activity were statistically significant for all four mental health outcomes (all *p* < 0.05). At the individual frequency-category level, compared with no vigorous physical activity, participation on 1–2 days was associated with lower odds of suicidal ideation (aOR = 0.875, 95% CI: 0.811–0.944), suicide plans (aOR = 0.810, 95% CI: 0.720–0.912), and suicide attempts (aOR = 0.803, 95% CI: 0.688–0.937). However, higher frequencies of vigorous physical activity were associated with higher odds of depressive symptoms, particularly at 3–4 days (aOR = 1.124, 95% CI: 1.058–1.193) and 5–7 days (aOR = 1.259, 95% CI: 1.177–1.347).

The overall tests for muscle-strengthening exercise were statistically significant for depressive symptoms, suicide plans, and suicide attempts, but not for suicidal ideation. At the individual frequency-category level, compared with no muscle-strengthening exercise, higher odds of depressive symptoms were observed at 1–2 days (aOR = 1.073, 95% CI: 1.017–1.132), 3–4 days (aOR = 1.230, 95% CI: 1.147–1.318), and 5–7 days (aOR = 1.260, 95% CI: 1.168–1.359). The odds of suicide attempts were also higher across these frequency categories, with the largest estimate observed at 3–4 days (aOR = 1.553, 95% CI: 1.291–1.868).

The overall tests for active transportation were statistically significant for all four mental health outcomes. At the individual frequency-category level, compared with no active transportation, participation on 5–7 days was associated with lower odds of depressive symptoms (aOR = 0.906, 95% CI: 0.861–0.954), whereas participation on 1–2 and 3–4 days was associated with higher odds of suicide attempts (aOR = 1.436, 95% CI: 1.137–1.813 and aOR = 1.452, 95% CI: 1.161–1.816, respectively).

The overall tests for physical education class participation were also statistically significant for all four mental health outcomes. At the individual frequency-category level, compared with no participation, two classes per week were associated with lower odds of depressive symptoms (aOR = 0.927, 95% CI: 0.866–0.992), suicidal ideation (aOR = 0.817, 95% CI: 0.744–0.898), suicide plans (aOR = 0.810, 95% CI: 0.702–0.936), and suicide attempts (aOR = 0.737, 95% CI: 0.618–0.878). Lower odds of suicidal ideation, suicide plans, and suicide attempts were also observed for participation in ≥3 classes per week. Overall, the adjusted associations varied across physical activity domains and mental health outcomes rather than showing a uniform pattern across increasing frequencies of physical activity.

## 4. Discussion

The present study examined the associations between different domains of physical activity and mental health outcomes among Korean adolescents using data from the 2025 Korea Youth Risk Behavior Survey. After accounting for the complex survey design and adjusting for sex, grade, academic achievement, household economic status, living arrangement, and weight status, the associations varied substantially according to the type of physical activity and mental health outcome. Vigorous physical activity and participation in physical education classes were generally associated with lower odds of several suicide-related outcomes, whereas ≥60 min of physical activity and muscle-strengthening exercise showed higher odds of depressive symptoms at several frequency levels. Active transportation showed mixed associations across frequency categories and outcomes. Recent evidence further suggests that active school transportation is influenced by psychosocial factors, including perceived competence, attitudes, social support, and social norms, indicating that active transportation occurs within a broader social and environmental context (Savolainen et al., 2024). These findings suggest that the relationship between physical activity and adolescent mental health cannot be characterized solely by overall participation frequency and may differ according to the domain and context of physical activity.

Analysis of general characteristics showed a tendency toward higher levels of perceived stress among students in higher grades and those with lower academic achievement and household economic status. This finding suggests that adolescent mental health may be associated with not only individual health behaviors but also academic burden and socioeconomic circumstances. Academic burden has been identified as an important factor associated with depression, anxiety, and suicide-related problems among adolescents (Han & Song, 2021; Steare et al., 2023). Studies on Korean adolescents also reported significant associations between stress arising from academic performance and family environments and suicidal ideation and suicide attempts (Im et al., 2017; Kim et al., 2024). Furthermore, the present finding that the prevalence of depressive symptoms and all suicide-related indicators differed according to grade level indicates that grade-level differences should be considered when interpreting adolescent mental health patterns. These differences may reflect variation in developmental stages, academic demands, and school environments, although the cross-sectional design of the present study does not allow these potential explanations to be directly evaluated. Accordingly, grade-specific contexts should be considered when interpreting the observed differences in adolescent mental health.

Analysis by type of physical activity revealed heterogeneous associations across mental health outcomes. Vigorous physical activity was significantly associated with all four mental health outcomes after adjustment for sex, grade, academic achievement, household economic status, living arrangement, and weight status. Compared with no vigorous physical activity, participation on 1–2 days was associated with lower odds of suicidal ideation, suicide plans, and suicide attempts, while participation on 3–4 days was associated with lower odds of suicidal ideation and suicide plans. These findings are broadly consistent with previous studies reporting inverse associations between physical activity and suicide-related outcomes among adolescents (Vancampfort et al., 2018; Rasmussen et al., 2025). However, the association with depressive symptoms showed a different pattern: vigorous physical activity on 3–4 and 5–7 days was associated with higher odds of depressive symptoms compared with no participation. Similarly, ≥60 min of physical activity and muscle-strengthening exercise were associated with higher odds of depressive symptoms across several frequency categories. These findings differ from previous studies reporting that higher levels of physical activity and exercise interventions are associated with reduced risk or fewer symptoms of depression among adolescents (Wang et al., 2022; Hou et al., 2025), despite proposed pathways involving physical fitness, sleep, self-efficacy, and social relationships (Rodriguez-Ayllon et al., 2019; Eo & Kim, 2023). Moreover, higher odds of suicide attempts were observed at some frequencies of ≥60 min of physical activity and muscle-strengthening exercise. Previous evidence has likewise suggested that exercise interventions may not consistently reduce suicide-related behaviors and that frequent vigorous physical activity may, in some contexts, be positively associated with suicide attempts (C. G. Lee et al., 2013; Fabiano et al., 2023). Taken together, these mixed findings indicate that the associations between physical activity and adolescent mental health may differ according to the type, frequency, and mental health outcome examined, and caution is warranted against interpreting physical activity as a uniformly protective factor.

Several factors may explain these contradictory findings. First, the measures of physical activity frequency used in this study did not fully capture the purpose, duration, setting, voluntary nature, or social context of the activities. Even at the same frequency, associations with mental health may differ depending on whether the physical activity is performed for leisure, athletic training, weight management, or emotional coping. It is also possible that adolescents experiencing mental health problems participated in physical activity more frequently to cope with depressive symptoms or stress, making the direction of the association difficult to determine. Although the multivariable models adjusted for sex, grade, academic achievement, household economic status, living arrangement, and weight status, residual confounding by unmeasured factors cannot be excluded. In addition, physical activity was assessed based on participation during the previous 7 days, whereas depressive symptoms and suicide-related outcomes referred to experiences during the previous 12 months. This mismatch in assessment periods may limit the extent to which current physical activity patterns can be directly linked to mental health experiences occurring over a substantially longer period. Therefore, the observed associations should be interpreted as cross-sectional relationships rather than evidence that particular frequencies or types of physical activity increase or decrease mental health problems.

The findings regarding active transportation and participation in physical education classes further demonstrate that associations with mental health may differ according to the context of physical activity. For active transportation, participation on 5–7 days per week was associated with lower odds of depressive symptoms compared with no participation, whereas participation on 1–2 and 3–4 days was associated with higher odds of suicide attempts. This mixed pattern differs in part from previous research reporting that active commuting by walking or cycling is associated with fewer depressive symptoms than passive commuting (Sun et al., 2015), suggesting that frequency alone may not fully capture the mental health correlates of active transportation. In contrast, participation in physical education classes showed more consistent associations with suicide-related outcomes. Compared with no participation, physical education classes were associated with lower odds of suicidal ideation across all participation frequencies, while lower odds of suicide plans and suicide attempts were observed at several frequency levels. Participation twice per week was also associated with lower odds of depressive symptoms, although the magnitude of this association was small (aOR = 0.927, 95% CI: 0.866–0.992) and should therefore be interpreted cautiously in terms of its practical significance. Physical education classes differ from individually chosen physical activities in that they are regularly provided within the school setting and offer opportunities for interaction with peers and teachers. The psychological and social benefits of participation in structured sports have also been reported (Eime et al., 2013). In addition, participation in physical education classes is associated with stress perception and suicidal ideation among Korean high school students and with experiences of sadness among adolescents in other countries (Brosnahan et al., 2004; S. Park et al., 2020). Consistent with the need for cautious interpretation, a recent systematic review found that the mental health and well-being outcomes associated with school-based physical education, physical activity, and sports were heterogeneous, highlighting the importance of considering the specific characteristics and context of school-based provision (Rocliffe et al., 2024). Such school-based physical activities may provide adolescents with regular opportunities for physical activity and social support (Andermo et al., 2020). However, some studies found no significant association between participation in physical education classes and suicide-related behaviors (Southerland et al., 2016), while bidirectional associations between sports participation and emotional and peer relationship problems were reported (Spiegler et al., 2024). Therefore, the present findings alone do not establish whether participation in physical education classes improves mental health; the potential roles of school adjustment, school attendance, health status, and peer relationships should also be considered.

The findings of this study suggest that adolescent mental health in relation to physical activity should not be considered solely in terms of increasing the overall amount of activity. Instead, the type, frequency, and context of physical activity may warrant consideration when interpreting associations with adolescent mental health. In particular, the relatively consistent associations observed for physical education participation with lower odds of several suicide-related outcomes suggest that structured school-based physical activity may represent a relevant setting for further investigation, although these findings should not be interpreted as evidence of a protective effect.

This study has several strengths, including the use of recent, large-scale national survey data, incorporation of the complex sampling design, examination of multiple physical activity domains, and adjustment for major sociodemographic characteristics and weight status. However, several limitations should be considered. First, the cross-sectional design precludes determination of temporal sequence or causality. Second, all variables were self-reported and may be subject to recall and social desirability bias; moreover, depressive symptoms and suicide-related indicators were assessed using single-item questions and should not be interpreted as clinical diagnoses. Third, physical activity was assessed over the previous 7 days, whereas mental health outcomes referred to the previous 12 months, limiting temporal comparability. Fourth, although sex and grade were adjusted for, sex- or school-level-specific interaction or stratified analyses were not performed, and potential heterogeneity across these groups may not have been fully captured. Fifth, the physical activity domains were analyzed in separate regression models and were not mutually adjusted for one another. Because these behaviors may overlap within individuals, the observed associations should be interpreted as domain-specific associations rather than as independent effects of each physical activity behavior. In addition, given the number of statistical comparisons, the findings should be considered exploratory and interpreted cautiously, particularly for isolated significant estimates. Sixth, the complete-case approach may have introduced selection bias if participants with missing data differed systematically from those included in the analysis. Finally, despite adjustment for major covariates, residual confounding by unmeasured factors cannot be excluded. Future longitudinal studies using more detailed measures are needed to clarify the temporal direction of these associations.

## 5. Conclusions

This study analyzed the associations between physical activity and mental health status among Korean middle and high school students using data from the 21st Korea Youth Risk Behavior Survey (KDCA, 2025). After adjustment for sex, grade, academic achievement, household economic status, living arrangement, and weight status, the direction of the associations differed across physical activity types and mental health outcomes. Overall, the adjusted analyses demonstrated heterogeneous associations across physical activity domains and mental health outcomes, indicating that these relationships should be interpreted according to the type and context of physical activity rather than the amount of physical activity alone.

These findings highlight the importance of considering differences in physical activity type and context when examining their relationships with adolescent mental health. Rather than uniformly expanding physical activity participation, structured settings such as physical education classes may warrant further investigation in relation to adolescent mental health. However, because this study used cross-sectional data and self-reported responses, causal relationships between physical activity and mental health could not be established. The different assessment periods for physical activity and mental health outcomes and the possibility of residual confounding should also be considered. Future longitudinal and intervention studies are needed to further examine the associations between specific types of physical activity and mental health and clarify their temporal relationships and practical significance.

## Figures and Tables

**Table 1:** General characteristics.

Variables		*N* (Weighted %)
Sex	Male	27,018 (51.3)
	Female	25,600 (48.7)
Grade	Grade 7	9433 (17.5)
	Grade 8	9167 (17.0)
	Grade 9	8951 (16.8)
	Grade 10	8523 (15.9)
	Grade 11	8360 (16.1)
	Grade 12	8184 (16.6)
Living arrangement	Living with family	50,065 (95.7)
	Living with relatives	269 (0.5)
	Boarding or living alone	293 (0.6)
	Dormitory	1830 (2.8)
	Residential care facility	161 (0.4)
Weight status	Underweight	3673 (7.1)
	Normal weight	37,074 (71.1)
	Overweight	4974 (9.2)
	Obesity	6897 (12.6)
Academic achievement	High	6297 (12.2)
	Upper-middle	13,605 (25.9)
	Middle	15,286 (28.9)
	Lower-middle	12,433 (23.4)
	Low	4997 (9.6)
Economic status	High	5703 (11.3)
	Upper-middle	16,587 (32.0)
	Middle	24,583 (46.0)
	Lower-middle	4834 (8.9)
	Low	911 (1.8)
	Total	52,618 (100.0)

**Table 2:** Health-related indicators by general characteristics.

Variables		*N* (%)	Positive Self-Rated Health (%)	Overweight Body Perception (%)	Perceived Stress (%)	Sufficient Sleep Recovery (%)
Grade	Grade 7	9433 (17.5)	73.6	36.9	36.9	36.3
	Grade 8	9167 (17.0)	70.3	36.6	41.6	27.0
	Grade 9	8951 (16.8)	68.8	36.7	41.1	22.1
	Grade 10	8523 (15.9)	68.3	38.6	41.0	17.0
	Grade 11	8360 (16.1)	65.9	39.7	43.5	16.6
	Grade 12	8184 (16.6)	64.0	38.8	43.0	15.8
	adjusted *F*		33.084 ***	4.891 ***	12.658 ***	234.944 ***
Living arrangement	Living with family (a)	50,065 (95.7)	68.7	37.8	40.9	22.9
	Living with relatives (b)	269 (0.5)	63.6	40.0	45.9	22.9
	Boarding or living alone (c)	293 (0.6)	63.7	32.5	43.4	21.7
	Dormitory (d)	1830 (2.8)	66.4	40.0	46.0	14.1
	Residential care facility (e)	161 (0.4)	63.9	34.6	44.5	26.4
	adjusted *F*		1.684	1.267	2.600	10.437 ***
Weight status	Underweight (a)	3673 (7.1)	61.6	0.8	42.4	21.3
	Normal weight (b)	37,074 (71.1)	71.8	24.6	40.2	22.1
	Overweight (c)	4974 (9.2)	66.3	87.9	43.6	24.3
	Obesity (d)	6897 (12.6)	55.7	96.6	43.6	25.5
	adjusted *F*		207.232 ***	6065.721 ***	12.814 ***	12.673 ***
Academic achievement	High (a)	6297 (12.2)	74.8	31.5	40.1	28.7
	Upper-middle (b)	13,605 (25.9)	71.1	35.7	40.2	23.6
	Middle (c)	15,286 (28.9)	70.2	36.5	38.5	22.9
	Lower-middle (d)	12,433 (23.4)	64.4	42.8	42.9	19.7
	Low (e)	4997 (9.6)	58.6	43.8	48.5	19.5
	adjusted *F*		103.967 ***	71.532 ***	34.553 ***	46.687 ***
Economic status	High (a)	5703 (11.3)	79.1	33.1	38.0	32.1
	Upper-middle (b)	16,587 (32.0)	73.0	36.0	39.1	25.0
	Middle (c)	24,583 (46.0)	65.8	38.3	40.6	20.0
	Lower-middle (d)	4834 (8.9)	56.4	46.4	52.1	17.9
	Low (e)	911 (1.8)	51.5	48.5	60.2	15.8
	adjusted *F*		187.318 ***	57.385 ***	82.491 ***	113.499 ***

Note: Values are survey-weighted percentages. Percentages represent the proportion classified as “Yes” for each health-related indicator. Adjusted *F* and *p*-values were obtained from Rao–Scott adjusted tests accounting for sampling weights, stratification, and clustering. *** *p* < 0.001.

**Table 3:** Mental health status according to grade level.

Variables	Grade	Yes	No	Adjusted *F*
		*n* (%)	*n* (%)	
Depressive symptoms	Grade 7	2229 (23.8)	7204 (76.2)	7.496 ***
	Grade 8	2419 (26.8)	6748 (73.2)	
	Grade 9	2467 (27.5)	6484 (72.5)	
	Grade 10	2034 (23.8)	6489 (76.2)	
	Grade 11	2164 (25.6)	6196 (74.4)	
	Grade 12	2075 (25.3)	6109 (74.7)	
Suicidal ideation	Grade 7	995 (10.9)	8438 (89.1)	16.242 ***
	Grade 8	1200 (13.4)	7967 (86.6)	
	Grade 9	1195 (13.6)	7756 (86.4)	
	Grade 10	808 (9.6)	7715 (90.4)	
	Grade 11	915 (10.9)	7445 (89.1)	
	Grade 12	806 (9.8)	7378 (90.2)	
Suicide plan	Grade 7	395 (4.3)	9038 (95.7)	9.136 ***
	Grade 8	460 (5.1)	8707 (94.9)	
	Grade 9	447 (5.1)	8504 (94.9)	
	Grade 10	302 (3.6)	8221 (96.4)	
	Grade 11	314 (3.7)	8046 (96.3)	
	Grade 12	299 (3.5)	7885 (96.5)	
Suicide attempt	Grade 7	250 (2.6)	9183 (97.4)	4.581 ***
	Grade 8	274 (2.9)	8893 (97.1)	
	Grade 9	245 (2.8)	8706 (97.2)	
	Grade 10	176 (2.0)	8347 (98.0)	
	Grade 11	202 (2.4)	8158 (97.6)	
	Grade 12	160 (1.9)	8024 (98.1)	

Note. Values are presented as unweighted n (survey-weighted %). Percentages represent within-grade row percentages. Adjusted *F* and *p*-values were obtained from Rao–Scott adjusted tests accounting for sampling weights, stratification, and clustering. *** *p* < 0.001.

**Table 4:** Distribution of mental health status across physical activity frequency categories.

Variables		*N* (%)	Depressive Symptoms		Suicidal Ideation		Suicide Plan		Suicide Attempt	
			Yes	No	Yes	No	Yes	No	Yes	No
Days of ≥60 min of physical activity (days/week)	0 days (a)	17,030 (32.9)	4152 (24.5)	12,878 (75.5)	2048 (12.4)	14,982 (87.6)	731 (4.4)	16,299 (95.6)	390 (2.3)	16,640 (97.7)
	1–2 days (b)	15,291 (29.0)	3923 (25.8)	11,368 (74.2)	1727 (11.1)	13,564 (88.9)	630 (4.0)	14,661 (96.0)	382 (2.4)	14,909 (97.6)
	3–4 days (c)	11,281 (21.3)	2919 (25.9)	8362 (74.1)	1199 (10.6)	10,082 (89.4)	481 (4.4)	10,800 (95.6)	290 (2.7)	10,991 (97.3)
	5–7 days (d)	9016 (16.8)	2394 (26.6)	6622 (73.4)	945 (10.8)	8071 (89.2)	375 (4.2)	8641 (95.8)	245 (2.7)	8771 (97.3)
	adjusted *F*		4.329 **		7.420 ***		1.034		2.144	
Days of vigorous physical activity (days/week)	0 days (a)	17,138 (33.1)	4390 (25.7)	12,748 (74.3)	2181 (13.0)	14,957 (87.0)	828 (4.9)	16,310 (95.1)	468 (2.7)	16,670 (97.3)
	1–2 days (b)	14,954 (28.4)	3708 (24.8)	11,246 (75.2)	1628 (10.8)	13,326 (89.2)	593 (3.8)	14,361 (96.2)	331 (2.1)	14,623 (97.9)
	3–4 days (c)	11,410 (21.5)	2861 (25.2)	8549 (74.8)	1164 (10.1)	10,246 (89.9)	454 (4.0)	10,956 (96.0)	276 (2.4)	11,134 (97.6)
	5–7 days (d)	9116 (17.0)	2429 (26.7)	6687 (73.3)	946 (10.8)	8170 (89.2)	342 (3.8)	8774 (96.2)	232 (2.6)	8884 (97.4)
	adjusted *F*		3.162 *		18.727 ***		8.313 ***		3.868 **	
Days of muscle-strengthening exercise (days/week)	0 days (a)	26,533 (50.9)	6763 (25.6)	19,770 (74.4)	3179 (12.2)	23,354 (87.8)	1119 (4.3)	25,414 (95.7)	615 (2.3)	25,918 (97.7)
	1–2 days (b)	13,195 (24.7)	3302 (25.0)	9893 (75.0)	1448 (10.9)	11,747 (89.1)	547 (5.1)	12,648 (94.9)	343 (2.5)	12,852 (97.5)
	3–4 days (c)	6976 (13.3)	1778 (25.7)	5198 (74.3)	736 (10.8)	6240 (89.2)	311 (4.9)	6665 (95.1)	197 (3.0)	6779 (97.0)
	5–7 days (d)	5914 (11.1)	1545 (26.0)	4369 (74.0)	556 (9.5)	5358 (90.5)	240 (3.9)	5674 (96.1)	152 (2.5)	5762 (97.5)
	adjusted *F*		0.837		11.104 ***		0.878		4.074 **	
Days of active transportation (days/week)	0 days (a)	19,065 (35.2)	5036 (26.6)	14,029 (73.4)	2158 (11.6)	16,907 (88.4)	829 (4.3)	18,236 (95.7)	483 (2.4)	18,582 (97.6)
	1–2 days (b)	3497 (6.5)	907 (26.4)	2590 (73.6)	386 (11.1)	3111 (88.9)	170 (5.1)	3327 (94.9)	108 (3.4)	3389 (96.6)
	3–4 days (c)	3959 (7.4)	1066 (27.3)	2893 (72.7)	485 (12.7)	3474 (87.3)	186 (4.9)	3773 (95.1)	132 (3.4)	3827 (96.6)
	5–7 days (d)	26,097 (50.9)	6379 (24.3)	19,718 (75.7)	2890 (11.1)	23,207 (88.9)	1032 (3.9)	25,065 (96.1)	584 (2.2)	25,513 (97.8)
	adjusted *F*		9.978 ***		2.459		4.162 **		8.926 ***	
Physical education classes (times/week)	0 times (a)	8526 (16.1)	2296 (27.3)	6230 (72.7)	1130 (13.6)	7396 (86.4)	422 (5.0)	8104 (95.0)	271 (3.1)	8255 (96.9)
	1 time (b)	9863 (18.8)	2548 (25.6)	7315 (74.4)	1077 (10.9)	8786 (89.1)	414 (4.2)	9449 (95.8)	241 (2.4)	9622 (97.6)
	2 times (c)	15,401 (29.9)	3746 (24.3)	11,655 (75.7)	1618 (10.5)	13,783 (89.5)	589 (3.8)	14,812 (96.2)	337 (2.1)	15,064 (97.9)
	≥3 times (d)	18,828 (35.2)	4798 (25.6)	14,030 (74.6)	2094 (11.4)	16,734 (88.6)	792 (4.3)	18,036 (95.7)	458 (2.5)	18,370 (97.5)
	adjusted *F*		6.676 ***		12.879 ***		5.283 **		6.614 ***	

Note. Values are presented as unweighted n (survey-weighted %). Percentages represent within-physical-activity-category row percentages. Adjusted *F* and *p*-values were obtained from Rao–Scott adjusted tests accounting for sampling weights, stratification, and clustering. * *p* < 0.05; ** *p* < 0.01; *** *p* < 0.001.

**Table 5:** Adjusted Associations Between Physical Activity Frequency and Mental Health Outcomes.

Variables	Frequency	Depressive Symptoms aOR (95% CI)	Suicidal Ideation aOR (95% CI)	Suicide Plan aOR (95% CI)	Suicide Attempt aOR (95% CI)
Days of ≥60 min of physical activity	0 days	1.000 (Ref.)	1.000 (Ref.)	1.000 (Ref.)	1.000 (Ref.)
	1–2 days	1.140 (1.080–1.203)	0.932 (0.864–1.007)	0.942 (0.832–1.066)	1.108 (0.938–1.308)
	3–4 days	1.231 (1.155–1.312)	0.938 (0.859–1.025)	1.064 (0.929–1.218)	1.235 (1.037–1.469)
	5–7 days	1.329 (1.241–1.423)	1.029 (0.935–1.133)	1.066 (0.913–1.245)	1.304 (1.071–1.587)
	Overall	*F* = 26.489 ***	*F* = 2.323	*F* = 1.253	*F* = 2.975 *
Days of vigorous physical activity	0 days	1.000 (Ref.)	1.000 (Ref.)	1.000 (Ref.)	1.000 (Ref.)
	1–2 days	1.047 (0.986–1.112)	0.875 (0.811–0.944)	0.810 (0.720–0.912)	0.803 (0.688–0.937)
	3–4 days	1.124 (1.058–1.193)	0.836 (0.766–0.912)	0.837 (0.729–0.961)	0.918 (0.766–1.099)
	5–7 days	1.259 (1.177–1.347)	0.958 (0.870–1.055)	0.802 (0.682–0.943)	0.975 (0.806–1.180)
	Overall	*F* = 17.156 ***	*F* = 7.246 ***	*F* = 5.199 **	*F* = 3.028 *
Days of muscle-strengthening exercise	0 days	1.000 (Ref.)	1.000 (Ref.)	1.000 (Ref.)	1.000 (Ref.)
	1–2 days	1.073 (1.017–1.132)	0.979 (0.905–1.059)	1.081 (0.960–1.217)	1.177 (1.004–1.379)
	3–4 days	1.230 (1.147–1.318)	1.108 (1.002–1.225)	1.312 (1.121–1.536)	1.553 (1.291–1.868)
	5–7 days	1.260 (1.168–1.359)	0.986 (0.883–1.102)	1.144 (0.955–1.370)	1.304 (1.052–1.616)
	Overall	*F* = 19.547 ***	*F* = 1.822	*F* = 3.874 **	*F* = 7.663 ***
Days of active transportation	0 days	1.000 (Ref.)	1.000 (Ref.)	1.000 (Ref.)	1.000 (Ref.)
	1–2 days	1.005 (0.913–1.107)	0.960 (0.846–1.090)	1.213 (0.994–1.482)	1.436 (1.137–1.813)
	3–4 days	1.067 (0.973–1.169)	1.146 (1.016–1.293)	1.160 (0.963–1.397)	1.452 (1.161–1.816)
	5–7 days	0.906 (0.861–0.954)	0.951 (0.885–1.022)	0.898 (0.803–1.004)	0.920 (0.807–1.050)
	Overall	*F* = 8.568 ***	*F* = 3.604 *	*F* = 5.101 **	*F* = 8.840 ***
Physical education classes	0 times	1.000 (Ref.)	1.000 (Ref.)	1.000 (Ref.)	1.000 (Ref.)
	1 time	0.961 (0.891–1.038)	0.840 (0.758–0.930)	0.907 (0.768–1.071)	0.815 (0.670–0.990)
	2 times	0.927 (0.866–0.992)	0.817 (0.744–0.898)	0.810 (0.702–0.936)	0.737 (0.618–0.878)
	≥3 times	1.008 (0.940–1.081)	0.823 (0.748–0.905)	0.817 (0.704–0.949)	0.774 (0.648–0.926)
	Overall	*F* = 2.669 *	*F* = 7.415 ***	*F* = 3.469 **	*F* = 4.498 **

Note. Values are presented as aORs (95% CIs). Models accounted for the complex survey design and were adjusted for sex, grade, academic achievement, household economic status, living arrangement, and weight status. Ref., reference category. * *p* < 0.05; ** *p* < 0.01; *** *p* < 0.001.

## Data Availability

Publicly available datasets were analyzed in this study. The 2025 Korea Youth Risk Behavior Survey raw data are available from the Korea Disease Control and Prevention Agency through the Korea Youth Risk Behavior Survey website (https://www.kdca.go.kr/yhs/, accessed on 15 July 2026). Access to the raw data requires agreement to the user information collection terms before download.
